# Pial arteriovenous fistula with a large intraparenchymal hemorrhage in a 9-year-old child: a case report and case-based mini review

**DOI:** 10.1007/s00381-025-06784-7

**Published:** 2025-03-17

**Authors:** Manina M. Etter, Raphael Guzman, Marios-Nikos Psychogios, Jehuda Soleman

**Affiliations:** 1 Department of Neurosurgery, University Hospital of Basel, Spitalstrasse 21, 4031 Basel, Switzerland; 2 Division of Pediatric Neurosurgery, University Children’s Hospital of Basel, Basel, Switzerland; 3 Faculty of Medicine, University Hospital of Basel, Basel, Switzerland; 4 Department of Radiology, Division of Diagnostic and Interventional Neuroradiology, University Hospital of Basel, Basel, Switzerland

**Keywords:** Pial arteriovenous fistula, Arteriovenous malformation, Cerebral angiography, Pediatric neurosurgery

## Abstract

**Background:**

Pial arteriovenous fistulas are rare cerebrovascular malformations, predominantly occurring in the pediatric population. The spectrum of symptoms is broad, ranging from incidental findings to intracranial hemorrhage. However, accurate diagnosis and optimal treatment require, among other factors, dynamic imaging modalities and interdisciplinary management.

**Case presentation:**

We describe a case of a 9-year-old patient presenting with acute spontaneous headache and apathy. MRI revealed a right temporal intraparenchymal hemorrhage, without signs of an underlying vascular pathology. Cerebral angiography was performed, revealing a suspected pial arteriovenous fistula. The patient was scheduled for surgical hematoma removal and resection of the pial fistula, with intraoperative angiographic control. After hematoma removal and resection of the pial fistula, intraoperative cerebral angiography revealed an additional fistula point that had not been appreciated on the initial preoperative angiography. The craniotomy was extended and the remaining fistula was resected. Final intraoperative angiography confirmed complete resection of the pial fistula.

**Conclusion:**

Pediatric pial arteriovenous fistulas are rare, complex, and challenging arteriovenous lesions. Accurate diagnosis and an interdisciplinary management are essential. However, consensus on the diagnostic workflow and treatment approach remains lacking. Therefore, we report our case and propose a diagnostic and therapeutic workup for ruptured vascular intracranial anomalies in children.

**Supplementary Information:**

The online version contains supplementary material available at 10.1007/s00381-025-06784-7.

## Introduction

Pial arteriovenous fistulas (pAVF) — also known as cerebral AVFs or non-galenic AVFs — are rare cerebrovascular anomalies, consisting of one or more dilated pial arteries directly connected to a cortical vein. Considered as a subtype of arteriovenous malformations (AVMs), pAVFs do not contain a subpial nidus intervening between the arterial and venous system [1, 2]. PAVFs differ from dural AVFs (dAVF) in terms of the fistula site and subsequent venous drainage patterns. The fistula site of a pAVF is located within the subpial meningeal space, whereas dAVF are characterized by an intradural fistula site. In contrast, galenic AVFs typically involve a persistent embryonic median prosencephalic vein [1].

The exact incidence and prevalence of pAVFs remain unknown. Previous studies suggest that pAVFs constitute approximately 1.6–7.3% of all intracranial vascular malformations, with a predominance in pediatric patients [3–7]. A recent study estimated the prevalence of pAVF to range from 0.1/ to 1 per 100′000, classifying it as a rare disease [8].

## Historical background

Due to their rarity and the challenges in describing their angioarchitecture before the advent of modern angiography, pAVFs were not initially recognized as a distinct entity among intracranial vascular malformations. In 1928, Walter Dandy classified pAVFs as “arteriovenous aneurysms” within the broader category of vascular malformations [9]. In 1945, Noran et al. introduced a classification system distinguishing “arteriovenous angioma” and “cerebral varices”; however, pAVFs did not precisely fit either category [10]. In 1966, McCormick revised the existing classification system, introducing the terms “varix” and “AVM” (also called angioma) but without a precise characterization of pAVFs [11].

Due to their rarity and the absence of a specific category in historical classification systems, pAVFs were often classified alongside AVMs. However, advances in cerebral angiography now allow for detailed characterization of pAVF angioarchitecture, providing a robust foundation for accurate diagnosis and tailored treatment strategies.

## Etiology and clinical presentation

PAVFs can occur at any age with a reported predominance in pediatric patients [3–7, 12]. In adults, pAVFs are most commonly acquired, often resulting from trauma [13], medical procedures [14–16], or following cerebral vein thrombosis[17]. In contrast, pediatric pAVFs are frequently congenital [5, 18, 19]. The exact pathophysiology of pAVF formation remains unclear, though proposed mechanisms include errors in vascular morphogenesis and disruptions in angiogenic growth factor pathways during embryogenesis [20, 21]. In children, pAVFs are often associated with congenital disorders, such as Klippel-Trenaunay-Weber syndrome and Rendu-Osler-Weber syndrome, also known as hemorrhagic hereditary telangiectasia (HHT), with the latter being the most frequently linked hereditary disease [22–26]. The genetic mutations most commonly implicated in vascular malformations involve the HHT genes (ENG, ACVRL, SMAD4) and RASA1.

The clinical presentation of pAVFs varies widely, ranging from asymptomatic incidental findings to acute intracranial hemorrhage, whereas pAVFs often become symptomatic in childhood, with symptomatology differing by age [8]. In children, common manifestations include developmental delay, macrocephaly, chronic hydrocephalus, and congestive heart failure, the latter being the most common presentation in neonates [8, 12, 27]. In adults, headaches, seizures, and focal neurological deficits are more prevalent [2, 28, 29]. The risk of intracerebral hemorrhage appears to increase with age, occurring more frequently in patients over 15 years [30].

## Diagnosis

In patients with acute symptoms, magnetic resonance imaging (MRI) with magnetic resonance angiography (MRA) and/or computed tomography (CT) with CT-angiography (CTA) are commonly used as initial diagnostic modalities [31–33]. These imaging techniques can also reveal indirect signs and complications of pAVFs, such as brain edema, intraparenchymal hemorrhage, or hydrocephalus [18, 34]. In some cases, hemorrhage and the resulting mass effect may obscure the underlying vascular malformation.

In recent years, conventional MRI and MRA have gained prominence in evaluating intracranial vascular malformations due to their non-invasive nature and superior delineation of surrounding structures. Compared to CT/CTA, MRI/MRA provides higher resolution for parenchymal structures and avoids radiation exposure, making it particularly advantageous for pediatric patients [32].

Detailed anatomic information is crucial, not only for the diagnosis but also for treatment planning. However, MRI/MRA and CT/CTA provide static images and cannot capture the hemodynamic characteristics of pAVFs, which are essential for treatment decisions and planning. Due to its superior spatial and temporal resolution, digital subtraction angiography (DSA) remains the gold standard for the definite diagnosis of pAVFs. DSA allows for precise delineation of the fistula site, arterial supply, and venous drainage as well as recording hemodynamic changes [32]. However, it is important to note that in case of an acute intraparenchymal hemorrhage, the angioarchitecture may be distorted, and an underlying pAVF or AVM nidus may be masked.

## Management

There is no established consensus on the treatment of (ruptured) pAVF in children. It is suggested that children with multiple cerebrovascular malformations may have genetic mutations associated with hereditary vascular diseases such as HHT. In such cases, genetic testing should be considered.

If left untreated, pAVFs are associated with a mortality rate of 63% [6, 35]. The primary treatment goal is the complete occlusion of the pAVF, which is typically achieved by disconnecting the fistula point. While vascular lesion resection is generally unnecessary, it may become essential in case of bleeding. This can often be accomplished by endovascular embolization, microsurgical disconnection, or a hybrid approach. Historically, before the development of modern endovascular techniques and embolic agents, cerebral AVFs were predominately managed through open surgical procedures. However, advancements in endovascular technology, coupled with increased expertise and the minimally invasive nature of endovascular interventions, have led to their widespread adoption, particularly in adult patients. However, ionizing radiation exposure poses a significant risk of long-term effects, particularly in the neonate. Therefore, endovascular treatment is often not the first-line therapeutic approach in pediatric patients, in contrast to adults. Studies have reported instances of treatment failure following endovascular procedures, necessitating additional therapeutic interventions [36, 37]. Further, pediatric cerebral AVFs differ from their adult counterparts, as they frequently present as high-flow lesions with anomalous vascular anatomy and tortuous feeding arteries. These characteristics increase the risk of iatrogenic injury during microcatheter navigation to the fistula site. Additionally, the small size of pediatric patients can further complicate arterial access and impose limitations on the volume of contrast agent administrated, as it must remain strictly to the maximum contrast dose of 4 cc/kg body weight [38–40]. In the case series of Madson et al., intervention-related hemorrhage following endovascular treatment of pAVFs was observed in 17 out of 135 cases, corresponding to an incidence of 12.6% [18]. Similarly, Mahmoud et al. reported a complication rate associated with this procedure and suggested that pAVF rupture may result from substantial hemodynamic changes following the occlusion of primary arterial feeders or impaired venous outflow [40].

However, open surgical intervention also presents significant risks, particularly in neonates, where limited blood loss and the frequent presence of congestive heart failure pose major challenges [18, 39]. Additionally, many of these vascular lesions are located in deep and eloquent brain regions, restricting both surgical access and visualization. The preoperative anatomy and angioarchitecture details delineated by DSA may be difficult to precisely correlate with the intraoperative field, complicating the identification of the fistula point [39]. This challenge is further exacerbated in cases of intraparenchymal hemorrhage, where visualization and identification of the target vessels and the fistula site are further impaired [32].

Given the inherited limitations of both open and endovascular approaches, a multidisciplinary strategy and multi-staged treatment regimen are often required [39]. However, in neonates and children, due to the aforementioned challenges, open surgical intervention is generally preferred over endovascular treatment, whenever feasible.

**Fig. 1:**
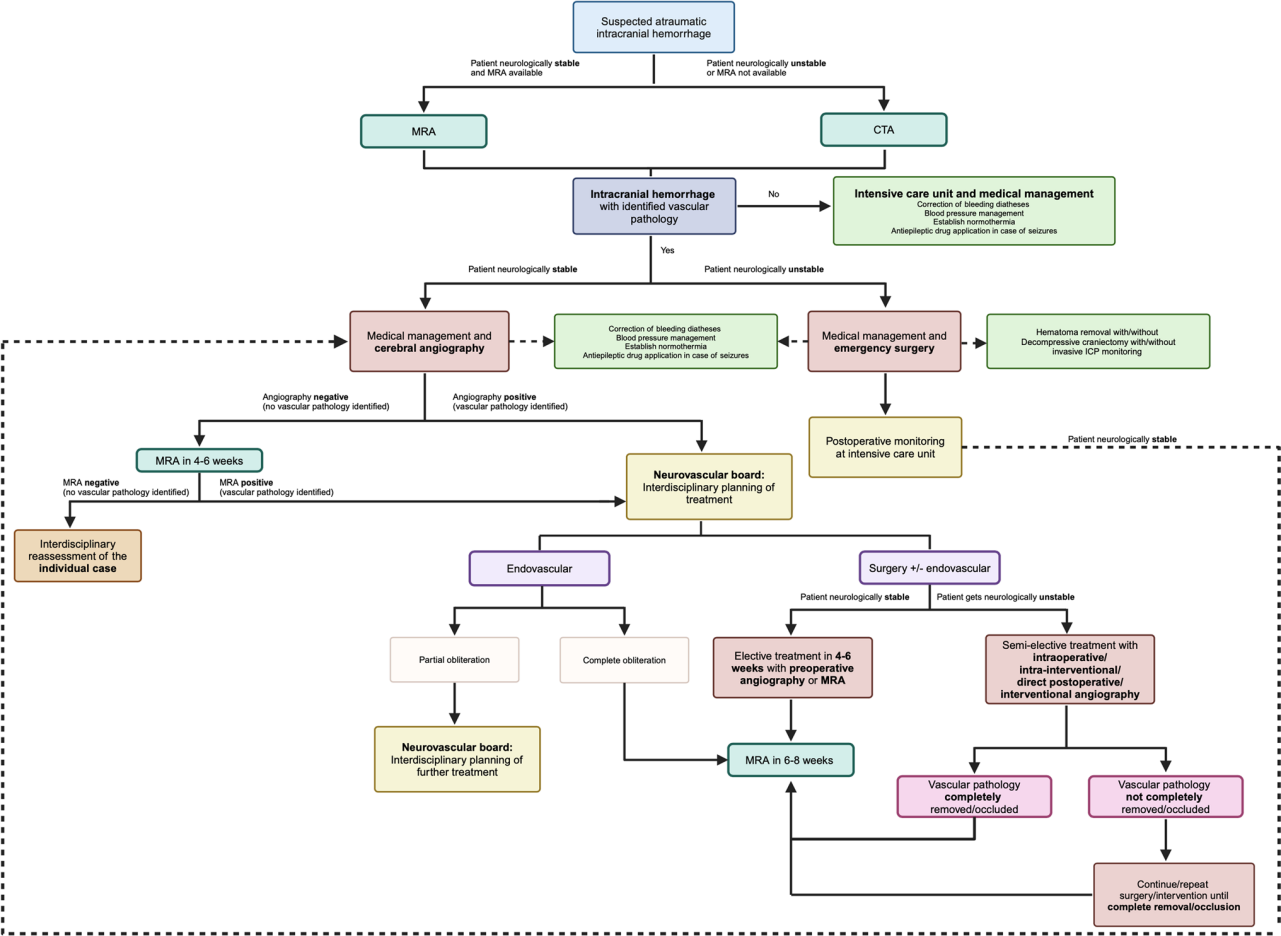
Flowchart showing a proposed workflow for the diagnostic workup and possible treatments for suspected intracranial hemorrhage in the pediatric population

Figure 1 presents a proposed a workflow for the diagnostic evaluation and treatment of children with suspected atraumatic intracranial hemorrhage. This workflow is based on a comprehensive review of the current literature and our institutional experience.

## Prognosis and outcome

The clinical outcome is significantly influenced by the complexity of the pAVF and the patients’ age at the time of presentation. A literature review by Madsen et al. reported excellent outcomes in the majority of patients, defined as the absence of neurological deficits at follow-up [18]. This finding is consistent with the retrospective, single institution results of Hetts et al., who observed a good outcome (modified Rankin Scale of 0–2) in 72% of their patients [8].

The treatment of pediatric pAVFs remains complex and presents several challenges that should not be overlooked. In general, younger patients (< 2 years old) are more likely to experience poor outcomes [8, 18]. This has been attributed to the frequent presence of congestive heart failure within this particular population [8, 18]. In addition to age at diagnosis, the literature review by Madsen et. al. identified intracranial hemorrhage at presentation as another indicator of increased mortality [18]. According to current literature, the most common procedure-related complications include intracranial hemorrhage and the development of neurological deficits [8, 18, 39].

## Exemplary case description

**Fig. 2:**
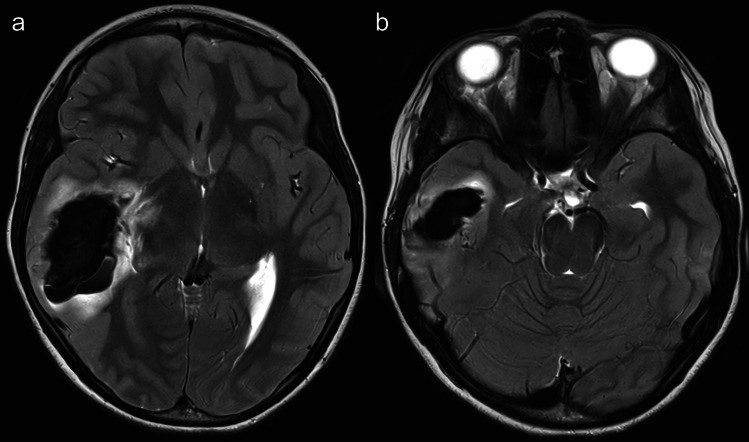
Axial T2-weighted MRI, performed during second presentation at our department, revealed a large intraparenchymal hemorrhage in the right temporal lobe with perilesional edema **a** at the level of the basal ganglia and internal capsule and **b** in the temporo-basal region

A 9-year-old female presented at our emergency department with acute headache accompanied with nausea and vomiting. Her symptoms improved following the administration of antiemetic medication, and she was discharged to return home. Two days later, she returned with progressive apathy and headache. Despite being drowsy, she did not lose consciousness and showed no signs of neurological deficits. Her medical history revealed no signs of trauma or preexisting conditions. MRI showed a large intraparenchymal hemorrhage (measuring 48 × 23 × 35 mm) (Fig. 2a, b) accompanied by perifocal edema in the right temporal lobe. However, due to the insufficient delineation of cerebrovascular structures on the MRI, we were unable to determine the presence or absence of any vascular malformation.

**Fig. 3:**
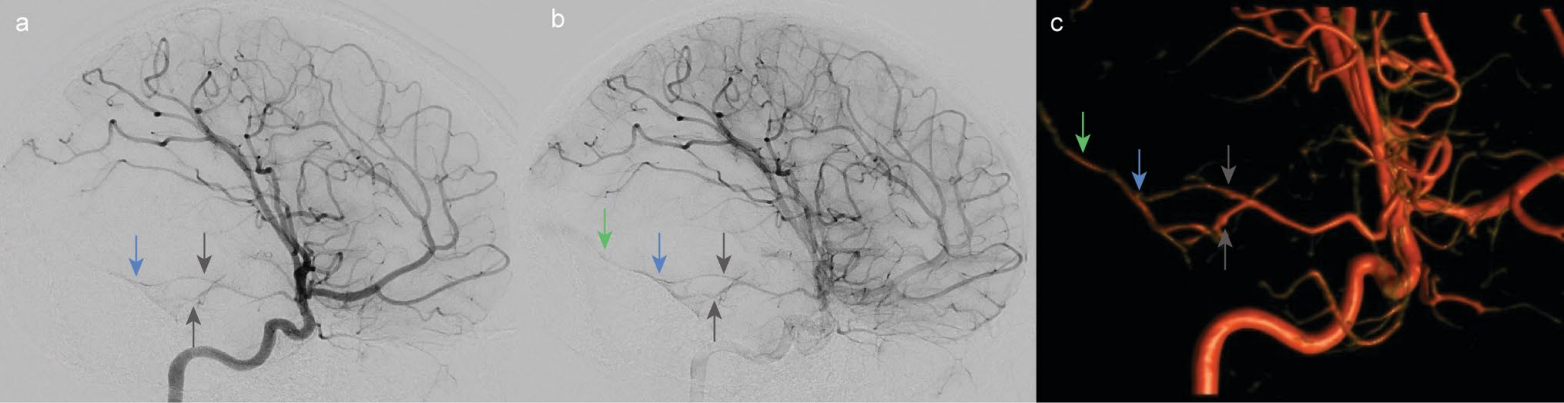
Diagnostic cerebral angiography performed on the second day of presentation revealed **a** a pAVF with feeding arteries (gray arrows) of the right temporal artery, arising from the MCA, in the early arterial phase of the angiography. The blue arrow indicates the fistula point. **b** Late arterial phase of the angiography depicting the pAVF with temporal feeding arteries (gray arrows), the fistula point (blue arrow), and the early draining vein (green arrow). **c** Volume-rendering technique (VRT) illustration of the pAVF with temporal feeding arteries (gray arrows), the fistula point (blue arrow), and the early draining vein (green arrow)

To identify the potential source of bleeding and assist in treatment planning, a DSA was performed (Fig. 3). The DSA images revealed a pAVF with feeding arteries originating from the right anterior temporal artery, the middle cerebral artery (MCA), and venous drainage into the right transverse sinus via a right temporal cortical vein. Notably, one of the primary differential diagnoses considered was a right temporal AVM, where a masked nidus might have been obscured by the extensive intraparenchymal hemorrhage.

The patient remained neurologically stable, and therefore, immediate neurosurgical intervention was deemed necessary. Consequently, we decided to admit her to the intensive care unit for observation. However, after several days of persistent headaches and vomiting, and following an interdisciplinary discussion, we opted to proceed with an elective craniotomy to obliterate the fistula and evacuate the intraparenchymal hemorrhage, which was likely the cause of her symptoms. The surgery was planned in our hybrid operating room angiography suite, which would allow us to verify the complete obliteration or resection of the vascular pathology during surgery. At this stage, a concealed AVM was still considered a plausible differential diagnosis.

**Fig. 4:**
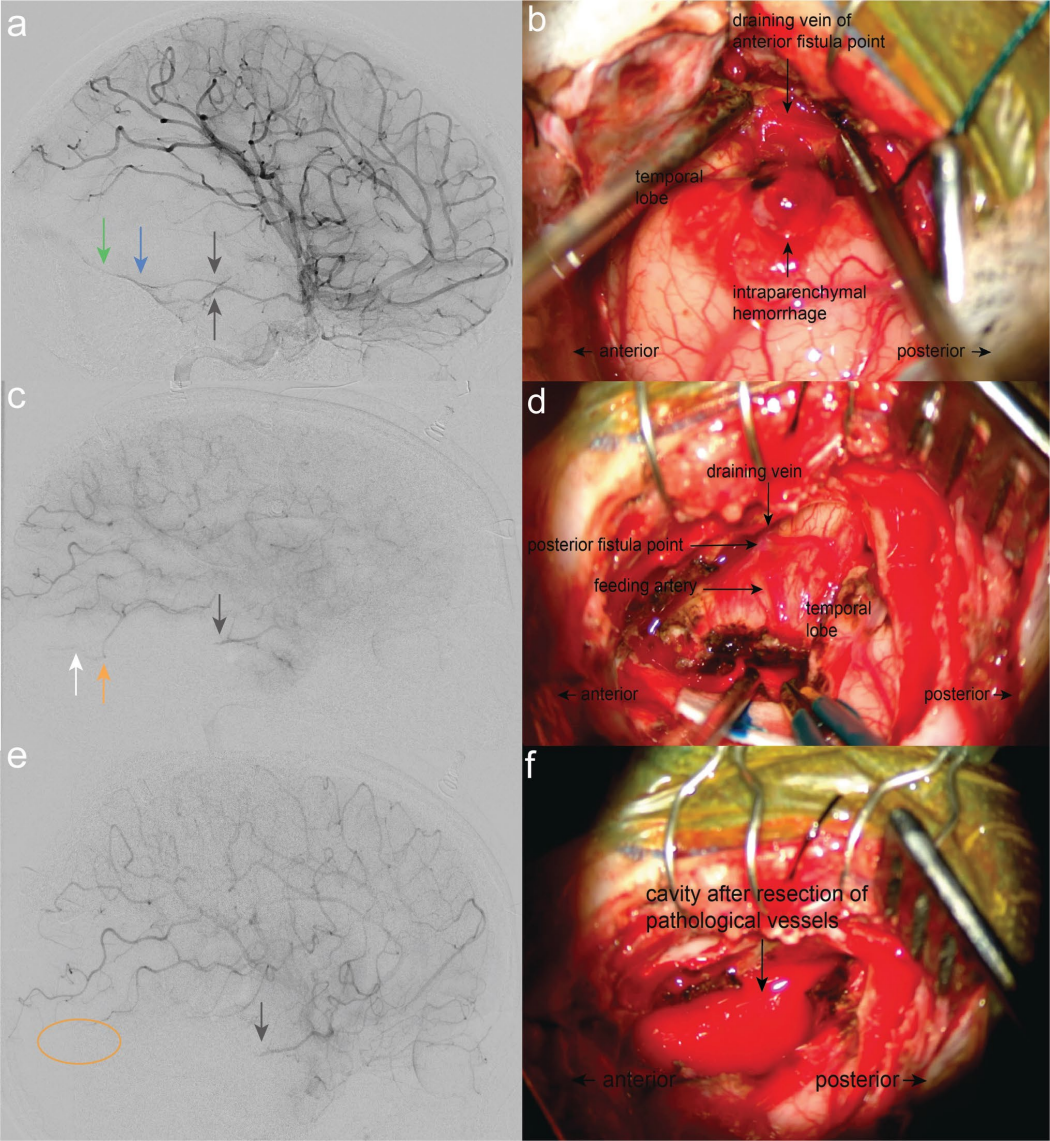
Preoperative DSA depicted **a** feeding arteries (gray arrows) arising from the temporal artery from the right MCA, the temporally located fistula point (blue arrow) and one draining vein (green arrow). **b** Intraoperative view before resection of the suspected fistula where the intraparenchymal hemorrhage masked the majority of pathological vessels, though one pathological arterialized vein was immediately identified. **c** First intraoperative DSA control showed occlusion of the feeding temporal arteries (gray arrow), but revealed a newly appearing, more posteriorly and caudally located fistula point (orange arrow) along with one draining vein (white arrow), which had likely been masked by the intraparenchymal hemorrhage. **d** Corresponding intraoperative finding after resection of the anteriorly located fistula point. **e** Final intraoperative DSA confirmed complete occlusion of both the anteriorly (gray arrow) and posterior-caudally (orange circle) located feeding arteries and the associated fistula point. **f** Surgical view after resection

A right temporo-basal craniotomy was performed under general anesthesia (Video 1). Upon dura incision, we immediately observed elevated intracranial pressure due to the swollen brain parenchyma. Mannitol was administered, and we initially evacuated the intraparenchymal hematoma which had extended to the surface of the right temporal lobe. After reducing approximately 75% of the hematoma volume, the brain relaxed, improving visualization of the pial vessels. Intraoperative DSA revealed two large afferent arteries (Fig. 4a, gray arrows) and an arterialized, early-draining vein (Fig. 4a, green arrow; Fig. 4b) draining into the right transverse sinus. Several small cortical arteries showed local oozing, which we controlled through coagulation and resection, ensuring local hemostasis. Intraoperative indocyanine green (ICG) angiography confirmed the presence of pial afferent arteries, an intervening nidus, and a draining arterialized vein (Video 1). With a clearer understanding of the fistula’s anatomy, we first obliterated the feeding arteries, while keeping the draining cortical vein intact as long as possible. Once the arterial feeders were secured, the draining vein was coagulated. Following removal and obliteration of the fistula, the hematoma was fully resected.

To confirm the complete resection of the fistula, intraoperative DSA was performed. The images revealed blood stasis in the feeding arteries arising from the right MCA without pathological venous drainage at the original fistula site (Fig. 4c, gray arrow; Fig. 4d). However, a new feeding artery and a more posteriorly located fistula point were identified (Fig. 4c, orange arrow; Fig. 4d) after obliteration of the suspected fistula — potentially due to the removal of the compressive hematoma and subsequent changes in hemodynamics. This newly discovered fistula had not been visible on the preoperative DSA and may have become apparent either due to the obliteration of the suspected fistula and subsequent changes in local hemodynamics or as a result of the removal of the compressive hematoma. Early venous drainage (Fig. 4c, white arrow; Fig. 4d) into the sinus remained present. As a result, we expanded the craniotomy posteriorly to explore the region harboring the newly identified fistula point. Additional ICG angiography confirmed the location of the posterior fistula point (Fig. 4d), which was subsequently obliterated and resected. Final intraoperative DSA and ICG confirmed the complete resection and obliteration of the pAVF (Fig. 4e, gray arrow and orange circle; Fig. 4f). After ensuring meticulous hemostasis, the dura was closed, the temporal bone was reinserted and secured with resorbable plates, while the skin was sutured in an ordinary fashion using resorbable stitches.

The postoperative course was uneventful. After one week of observation, the patient showed good wound healing and was discharged to her home without any neurological deficits.

## Conclusions

PAVFs are relatively rare, accounting for approximately 1.6–7.3% of all intracranial vascular malformations, mainly seen in children. Unlike dural AVFs, pAVFs occur in the subpial space, and lack an intervening nidus that connects the feeding arteries and draining veins. Pediatric pAVFs differ from their adult counterparts in terms of angioarchitecture, clinical presentation, and management. In children, pAVFs are often considered congenital, and genetic testing should be considered in selected cases [41]. While adults typically present with acute symptoms, children more commonly exhibit chronic symptoms such as developmental delay, hydrocephalus, or macrocephaly [5].

PAVFs carry a mortality rate of 63% if left untreated [6]. Therefore, accurate diagnosis and a clear understanding of the natural history of the condition are critical for successful treatment. It is essential to recognize the limitations, risks, and advantages of the various imaging modalities to ensure accurate diagnosis, facilitate effective treatment planning, and ultimately achieve favorable outcomes.

Despite recent advancements in endovascular and open surgical techniques that have significantly improved the management of AVMs and particularly pAVFs, an interdisciplinary approach remains crucial. According to the current literature, in most cases, an excellent outcome can be achieved through surgical resection or endovascular embolization or a combination of both treatments [4, 41].

Intraoperative DSA might be crucial to identify remaining arterial feeders after initial resection of the lesion, while the patient is still under general anesthesia. This might prevent the need for any further surgery, early-postoperative imaging, or potential additional intracranial hemorrhages. As demonstrated in this report, preoperative imaging can be particularly challenging in patients presenting with acute intraparenchymal hemorrhage. A precise understanding of the angioarchitecture and an accurate diagnosis are sometimes only achievable during surgery.

## Supplementary Information

Below is the link to the electronic supplementary material.

Supplementary file1 Video 1. Case description and surgery with intraoperative angiography control (MP4 488175 KB)

## Data Availability

No datasets were generated or analysed during the current study.
